# Audiological profile of patients treated for childhood cancer^☆^^☆☆^

**DOI:** 10.1016/j.bjorl.2015.11.021

**Published:** 2016-04-13

**Authors:** Patricia Helena Pecora Liberman, Maria Valéria Schmidt Goffi-Gomez, Christiane Schultz, Paulo Eduardo Novaes, Luiz Fernando Lopes

**Affiliations:** a Núcleo de Audiologia do A.C. Camargo Cancer Center, São Paulo, SP, Brazil; b Hospital Vitoria, Santos, SP, Brazil; c Hospital de Câncer de Barretos, Departamento de Pediatria, Barretos, SP, Brazil

**Keywords:** Radiotherapy, Ototoxicity, Chemotherapy, Cisplatin, Hearing loss, Hearing, Radioterapia, Ototoxicidade, Quimioterapia, Cisplatina, CDDP, Perda auditiva, Audição

## Abstract

**Objective:**

To characterize the hearing loss after cancer treatment, according to the type of treatment, with identification of predictive factors.

**Methods:**

Two hundred patients who had cancer in childhood were prospectively evaluated. The mean age at diagnosis was 6 years, and at the audiometric assessment, 21 years. The treatment of the participants included chemotherapy without using platinum derivatives or head and neck radiotherapy in 51 patients; chemotherapy using cisplatin without radiotherapy in 64 patients; head and neck radiotherapy without cisplatin in 75 patients; and a combined treatment of head and neck radiotherapy and chemotherapy with cisplatin in ten patients. Patients underwent audiological assessment, including pure tone audiometry, speech audiometry, and immittancemetry.

**Results:**

The treatment involving chemotherapy with cisplatin caused 41.9% and 47.3% hearing loss in the right and left ear, respectively, with a 11.7-fold higher risk of hearing loss in the right ear and 17.6-fold higher in the left ear *versus* patients not treated with cisplatin (*p* < 0.001 and *p* < 0.001, respectively). Children whose cancer diagnosis occurred after the age of 6 have shown an increased risk of hearing loss *vs*. children whose diagnosis occurred under 6 years of age (*p* = 0.02).

**Conclusion:**

The auditory feature found after the cancer treatment was a symmetrical bilateral sensorineural hearing loss. Chemotherapy with cisplatin proved to be a risk factor, while head and neck radiotherapy was not critical for the occurrence of hearing loss.

## Resumo

**Objetivo:**

Caracterizar as alterações auditivas após o tratamento do câncer, segundo o tipo de tratamento identificando os fatores preditivos.

**Método:**

Foram avaliados prospectivamente duzentos pacientes que tiveram cancer na infância. A idade média ao diagnóstico foi de 6 anos e à avaliação audiométrica de 21 anos de idade. O tratamento incluiu quimioterapia sem uso de derivados de platina ou radioterapia em cabeça e pescoço em 51 pacientes; quimioterapia com uso de cisplatina sem radioterapia em 64 pacientes; radioterapia em cabeça e pescoço sem cisplatina em 75 pacientes; e 10 pacientes receberam o tratamento combinado de radioterapia em cabeça e pescoço e quimioterapia com cisplatina. Os pacientes foram submetidos à avaliação audiológica incluindo audiometria tonal, audiometria vocal e imitanciometria.

**Resultados:**

O tratamento envolvendo quimioterapia com cisplatina levou a 41,9% e 47,3% de perda auditiva na orelha direita e esquerda, respectivamente, apresentando risco 11,7 vezes maior de desenvolver perda auditiva na orelha direita e 17,6 vezes na orelha esquerda do que aqueles que não receberam cisplatina (p < 0,001 e p < 0,001; respectivamente). Crianças cujo diagnóstico do câncer ocorreu após os 6 anos de idade mostraram maior risco de apresentar perda auditiva do que crianças menores do que 6 anos de idade (p = 0,02).

**Conclusão:**

A característica audiológica encontrada após tratamento oncológico foi perda auditiva sensorioneural bilateral simétrica. A quimioterapia com cisplatina mostrou ser fator de risco, enquanto a radioterapia em cabeça e pescoço não foi determinante para aquisição da perda auditiva.

## Introduction

Over the last two decades, childhood cancer mortality has decreased significantly; however, it still represents the second leading cause of death in Brazil.^1^ Currently, with the advances in diagnosis, improved treatments, and appropriate clinical support, an increase in the cure rate of malignancies in childhood is a possibility.^2^ In the face of an increasing survival rate, these individuals are now monitored for several years. Thus, it is possible to observe the impact of late effects of treatment on the quality of life of these young adults.

The use of different treatment modalities (surgery, radiotherapy, and chemotherapy) and the combination of these modalities contribute to improved results, both in controlling the disease and in improving survival rates.^3^

Among the ototoxic drugs, cisplatin is an antineoplastic agent with proven anti-tumor activity, but which may have ototoxicity as a side effect; the dose related to risk has been described as being 400 mg/m^2^.^2^, ^4^, ^5^

Head and neck radiotherapy concomitantly employed with cisplatin (cis-diamminedichloroplatinum [CDDP]) increases the likelihood of severe hearing loss.^6^, ^7^ However, when the drug is administered alone and in lower doses (50–60 Gy), no clinically significant hearing loss occurs.^8^, ^9^

Ototoxicity, *i.e.*, the effect represented by an injury to the peripheral organ of hearing, is characterized by an irreversible descending bilateral sensorineural hearing loss.^10^, ^11^ The incidence of the hearing loss is quite variable, due to the method of drug administration, tumor location, state of renal function, patient's age, associated drugs, radiotherapy, pre-existing hearing loss, cumulative dose, total dose of treatment, and individual susceptibility.^12^, ^13^

This study was conducted with the aim of characterizing the audiologic profile of patients who had cancer in childhood and were out of cancer treatment for at least eight years; to relate the hearing loss found with respect to the type of treatment and age; and to identify predictive factors for hearing loss.

## Methods

We prospectively evaluated children who had cancer treated between 2000 and 2004, and who had completed treatment for at least eight years, and who had been monitored in a group of pediatric studies on the late effects of cancer treatment. Patients with history of previous otologic disease or who had been submitted to a surgery involving the auditory system were excluded. The study was approved by the Research Ethics Committee of the institution under the protocol 549/03. Eligible patients or their legal guardians were consulted on the possibility of participating in the study, and were asked to sign the informed consent.

Patients were interviewed at the Pediatric Outpatient Clinic in order to investigate the presence of hearing complaints and then were referred to a hearing evaluation in the institution's Audiology Service, regardless of the presence of hearing complaints. Otoscopy was conducted before the test and, if the patient had cerumen or any suspicion and/or obstruction that prevented the test, he/she was referred to the otorhinolaryngologist before evaluation.

For hearing assessment, auditory quantification tests (pure tone audiometry and speech audiometry) and evaluation tests of the tympanic-ossicular system (immittancemetry) were performed. To this end, a Madsen Orbiter 922 audiometer and a Madsen Zodiac 901 immittancemeter were used.

The dose of CDDP received by the participants was calculated and adjusted by the pediatric oncologist for a body surface area of 1 m^2^. The clinical records of all patients who underwent head and neck radiotherapy were analyzed, taking into account the side on which radiotherapy was performed and whether the auditory system was included in the radiation field. The total dose and the estimated dose of radiation reaching the auditory system were calculated for each ear by a radiation oncologist, based on the planning form. The variable “radiation reaching the auditory system” was categorized as: no Rxt, Rxt ≤ 4000 cGy, and Rxt > 4000 cGy.^9^, ^14^

Patients were studied according to the type of treatment performed, based on the use of chemotherapy with CDDP or head and neck radiation therapy.

Hearing loss criteria were based on the Bureau International d’Audiophonologie – BIAP,^15^ which considers hearing loss as the presence of pure tone thresholds >20 dB in 0.5–4 kHz frequencies.

### Statistical analysis

To identify hearing loss predictors, a dichotomous variable (yes/no) was created, and hearing loss was diagnosed only in light of changes in the frequencies from 0.25 to 4 kHz. Hearing loss at 6 and 8 kHz was not included in the statistical analysis, due to the minor handicap that these losses cause in daily life.^16^, ^17^

The variable “age at diagnosis” was categorized as ≤6 years and >6 years, based on the median of the values found.

Measures of central tendency and of dispersion for quantitative variables and absolute and relative frequencies for categorical variables were calculated. In order to verify the association among independent variables and hearing loss, the associative chi-squared test or Fisher's exact test (when at least one of the expected frequencies was <5) was used. To identify independent risk factors for occurrence of hearing loss, logistic regression (with raw and adjusted odds ratios and their respective 95% confidence intervals) was used. For all statistical tests, an error *α* = 5% was established, *i.e.*, the results were considered statistically significant at *p* < 0.05.

## Results

The selected sample included 200 patients treated for childhood cancer, who were out of treatment during the study period. Table 1 shows the distribution according to the primary tumor and the type of treatment used. In this study, 51 participants did not undergo head and neck radiotherapy and were not medicated with CDDP, 64 received chemotherapy with CDDP and did not undergo head and neck radiotherapy, 75 underwent head and neck radiotherapy without chemotherapy with CDDP, and ten patients underwent head and neck radiotherapy and chemotherapy with CDDP. In most cases, children treated by Rxt without CDDP had a diagnosis of leukemia or retinoblastoma and were treated with megavoltage radiation. For patients who received CDDP + Rxt, the total radiation dose was higher (4214.0 ± 678.9 cGy) *vs.* patients who received only Rxt (2996.8 ± 1427.8 cGy) (Table 2).

**Table 1:** Distribution of patients, according to the type of primary cancer at diagnosis and treatment type (GEPETTO 2000–2004).

Malignant neoplasm	w/o Rxt w/o CDDP	CDDP	Rxt	Rxt + CDDP	Total
	*n* (%)	*n* (%)	*n* (%)	*n* (%)	*n* (%)
Bone tumor	8 (15.7)	39 (60.9)	1 (1.3)	0 (0.0)	48 (24.0)
Leukemias	1 (2.0)	0 (0.0)	43 (57.3)	0 (0.0)	44 (22.0)
Lymphomas (NHL, HL)	14 (27.5)	0 (0.0)	10 (13.3)	0 (0.0)	24 (12.0)
Retinoblastoma	8 (15.7)	1 (1.6)	10 (13.3)	5 (50.0)	24 (12.0)
Germ cell tumor	2 (3.9)	12 (18.7)	0 (0.0)	0 (0.0)	14 (7.0)
Kidney tumors	12 (23.5)	1 (1.6)	0 (0.0)	0 (0.0)	13 (6.5)
Soft tissue sarcomas	3 (5.8)	3 (4.6)	3 (4.0)	2 (20.0)	11 (5.5)
CNS tumor	0 (0.0)	0 (0.0)	4 (5.3)	2 (20.0)	6 (3.0)
SNS tumor (neuroblastoma)	1 (2.0)	4 (6.3)	0 (0.0)	1 (10.0)	6 (3.0)
Unspecified	0 (0.0)	1 (1.6)	4 (5.3)	0 (0.0)	5 (2.5)
Carcinomas	2 (3.9)	1 (1.6)	0 (0.0)	0 (0.0)	3 (1.5)
Liver tumors	0 (0.0)	2 (3.1)	0 (0.0)	0 (0.0)	2 (1.0)

Total	51 (100)	64 (100)	75 (100)	10 (100)	200 (100)

CNS, central nervous system; SNS, sympathetic nervous system; CDDP, cisplatin; Rxt, radiotherapy; NHL, non-Hodgkin's lymphoma; HL, Hodgkin's lymphoma.

**Table 2:** Means and standard deviations of the radiation dosage (Rxt) and of cisplatin (CDDP) used according to the type of treatment for the right (RE) and left (LE) ear (GEPETTO 2000–2004).

Type of treatment	*n*	Rxt total dose (cGy)	RE Rxt dose (cGy)	LE Rxt dose (cGy)	CDDP dose (mg/m^2^)
w/o Rxt, w/o CDDP	51	–	–	–	–
CDDP	64	–	–	–	647.4 ± 326.5
Rxt	75	2996.8 ± 1427.8	1894.8 ± 1544.3	1821.5 ± 1540.8	–
Rxt + CDDP	10	4214.0 ± 678.9	2292.0 ± 1744.2	1524.0 ± 1692.7	668.1 ± 260.7

Table 3 shows that 104 (52%) participants of the sample were male and 96 (48%) were female.

**Table 3:** Distribution of patients who met the hearing loss criteria in relation to the factors studied: sex, age, radiation, and chemotherapy with CDDP (GEPETTO 2000–2004).

	RE		Total	*p*^a^	LE		Total	*p*^a^
	w/o loss *n* (%)	With loss *n* (%)	*n*		w/o loss *n* (%)	With loss *n* (%)	*n*	
*Gender*				0.525				0.062
Male	86 (82.7)	18 (17.3)	104		88 (84.6)	16 (15.4)	104	
Female	76 (79.2)	20 (20.8)	96		71 (74.0)	25 (26.0)	96	

*Age*				**0.001**				**0.001**
≤6 years	100 (90.1)	11 (9.9)	111		98 (88.3)	13 (11.7)	105	
>6 years	62 (69.7)	27 (30.3)	89		61 (68.5)	28 (31.5)	95	

*Rxt*				**0.025**				**0.020**
w/o Rxt	103 (76.9)	31 (23.1)	134		103 (74.6)	35 (25.4)	138	
≤4000 cGy	52 (92.9)	4 (7.1)	56		50 (92.6)	4 (7.4)	54	
>4000 cGy	7 (70.0)	3 (30.0)	10		6 (75.0)	2 (25.0)	8	

*Chemo*				**<0.001**				**<0.001**
w/o CDDP	119 (94.4)	7 (5.6)	126		120 (95.2)	6 (4.8)	126	
With CDDP	43 (58.1)	31 (41.9)	74		39 (52.7)	35 (47.3)	74	

*Total*	162 (81.0)	38 (19.0)	200		159 (79.5)	41 (20.5)	200	

Bolded *p* refer to statistical significance (*p* < 0,05).

a Statistics according to the chi-squared test.

## Hearing loss characterization

**Figure 1:**
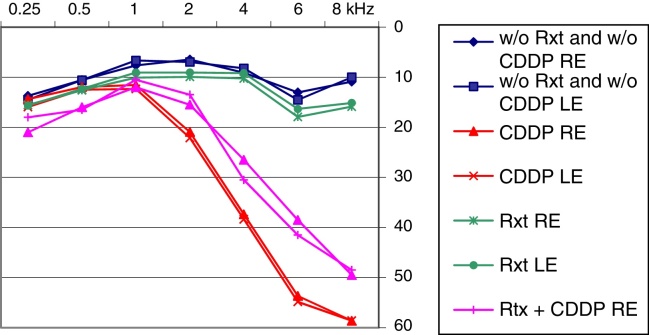
Mean audiometric configuration of hearing thresholds by type of treatment. CDDP, cisplatin; Rxt, radiation treatment; RE, right ear; LE, left ear.

Patients who received CDDP or CDDP + Rxt were affected by a symmetrical bilateral sensorineural hearing loss at 4-, 6-, and 8-kHz frequencies. Hearing loss did not occur in cancer patients who did not receive a risk-to-hearing treatment or head and neck Rxt as a single treatment (Fig. 1).

## Identification of predictive factors for hearing loss

In the comparison among the three groups without Rxt, and with Rxt up to 4000 or above 4000 cGy, both to the right (*p* = 0.025) and to the left (*p* = 0.020) side, a statistically significant difference was observed. It is not possible, however, to state that the Rxt factor has influenced the hearing loss because, the values for the percentage of hearing loss comparing the group that did not receive Rxt *vs.* those that received more than 4000 cGy are similar. Thus, it is believed that the statistical significance is due to the lower percentage of hearing loss in the group that received less than 4000 cGy, thereby differing from the other two groups, as can be seen in Table 3.

## Multivariate analysis of predictive factors for hearing loss

In the multivariate analysis (considering those patients who did not use CDDP as a control group), use of CDDP and age at the time of diagnosis were predictive factors for hearing loss. Children whose treatment included the use of CDDP showed an 11.7-fold increased risk for hearing loss in the right ear and a 17.6-fold increased risk in the left ear (*p* < 0.001 for both ears), compared to children who did not use CDDP. Diagnosis before the age of 6 years imparted a 2.7 times higher risk for hearing loss in the right ear (*p* = 0.02), compared with children aged ≤6 years (Table 4).

**Table 4:** Multiple analysis of predictive factors of hearing loss in the right (RE) and left (LE) ear.

Ear	Variables	Categories	Raw OR	Adjusted OR	95% CI adjusted OR	*p*
RE	CDDP	No	1.0	1.0	Reference	
		Yes	12.2	11.7	4.2; 32.1	<0.001^a^
	Rxt	w/o Rxt	1.0	1.0	Reference	
		≤4000 cGy	0.3	0.9	0.2; 3.3	0.894
		>4000 cGy	1.4	4.3	0.8; 24.1	0.196
	Age (years)	≤6	1.0	1.0	Reference	
		>6	3.9	2.7	1.1; 6.4	0.028
LE	CDDP	No	1.0	1.0	Reference	
		Yes	17.9	17.6	6.0; 51.4	<0.001^b^
	Rxt	w/o Rxt	1.0	1.0	Reference	
		≤4000 cGy	0.2	0.9	0.2; 3.4	0.912
		>4000 cGy	0.9	3.9	0.5; 31.2	0.192
	Age (years)	≤6	1.0	1.0	Reference	
		>6	3.5	2.1	0.9; 5.0	0.084

OR, odds ratio; CDDP, cisplatin; Rxt, radiotherapy.

a Hosmer–Lemeshow test (*p* = 0.856).

b Hosmer–Lemeshow test (*p* = 0.459).

In the multivariate analysis, the dose of radiotherapy was not a risk factor for hearing loss when patients who did not receive Rxt were used as a control group.

## Discussion

This study aimed to establish a relationship of hearing changes found by type of treatment and age, and to identify predictive factors of hearing loss in patients who had cancer in childhood and had completed cancer treatment several years before. To define which treatment implied risk of hearing loss, a separation was required between the ears, considering that the incidence of radiation varied with the tumor site.

This study found a predominance of exams with thresholds within the normal range in patients who did not undergo treatment with CDDP. Conversely, patients who used CDDP or CDDP + Rxt showed a predominance of bilateral symmetrical sensorineural hearing loss at 4-, 6-, and 8-kHz frequencies.^11^, ^12^, ^18^, ^19^, ^20^, ^21^, ^22^ It is possible to infer that hearing loss is related to the type of cancer treatment, even considering that these are individuals assessed many years after the end of their therapy, since the group whose treatment did not involve CDDP or Rxt, under the same conditions, showed no hearing loss.

Paulino et al.,^23^ Johannesen et al.,^24^ Low et al.,^8^ and Dell’Aringa et al.^9^ reported that doses between 4000 and 6000 cGy were risk dosages for hearing loss, and suggested audiological monitoring. Treatment with Rxt in head and neck tumors can cause other ear disorders such as otitis externa, serous otitis media,^25^ necrosis of the external auditory canal, and osteoradionecrosis of the temporal bone.^14^, ^24^, ^26^ In the present series, leukemias were prevalent in patients who were treated with Rxt without CDDP, and these individuals showed no hearing loss, which is consistent with the results reported in the study by Thibadoux et al.^27^ Indeed, the dosage of radiation penetrating the right (2292.0 ± 1744.2 cGy) and the left (1524.0 ± 1692.7 cGy) ear was of low-risk for all patients who underwent Rxt. Table 3 shows that 92.9% of patients who received doses of radiation therapy below 4000 cGy did not show hearing loss, justifying the significant association. In the multivariate analysis of predictive factors for hearing loss, Rxt was not a risk factor for hearing loss (Table 4).

Patients treated with CDDP and CDDP + Rxt received high doses of CDDP – higher than the dose considered a risk for hearing loss (≥400 mg/m^2^). In this series, the mean dose of CDDP in patients treated with chemotherapy was 650 mg/m^2^, and in individuals who received CDDP + Rxt, the dose was 670 mg/m^2^. Li et al.^22^ pointed out the relationship between dose of CDDP and hearing loss, with dosages ≥400 mg/m^2^ showing a higher risk for hearing loss. Studies using conventional frequencies (0.25–8 kHz) for hearing evaluation reveal a variation of 20–70% in the incidence of hearing loss.^28^ This change occurs due to several factors, including: the assessed frequencies, age of the individuals, dosage of CDDP, drug dosing schedule, and criteria used to define hearing loss. The present study found a prevalence of 41.9% and 47.3% for RE and LE, respectively, based on hearing loss criteria at frequencies from 0.25 to 4 kHz.

Li et al.^22^ indicated that there is a greater risk for hearing loss in children under 5 years of age. Brock et al.,^18^ Simon et al.,^29^ and Gunn et al.^30^ found no statistically significant relationship between the use of CDDP and age.

In the present sample, it was found that the age of 6 years at the time of cancer diagnosis imparted a 2.7 times higher risk for hearing loss (*p* = 0.02) compared with children ≤6 years of age at diagnosis, but only for the right ear, with a tendency for the left ear (OR = 2.1; *p* = 0.08). This finding may be due to this series of patients, who had their hearing loss concentrated in osteosarcoma diagnoses, more often established in adolescence (Table 1).

The present study found no statistically significant relationship for gender; however, Yancey et al.^28^ reported that gender and cumulative dose are the most important clinical markers of ototoxicity. The severity of ototoxicity may be inversely related to age at the time of treatment, and younger children have greater degrees of hearing loss after treatment.^28^ Ondrey et al.^31^ believe that the combination of these two treatments (Rxt + QT) will be the best cancer treatment in the future; however, both therapies cause ototoxic effects.

In this study, the sample of patients undergoing a combined treatment (Rxt + CDDP) was small (*n* = 10); but the same degree and type of hearing loss was found in patients who underwent chemotherapy with CDDP without Rxt. This finding demonstrates that, in this study, Rxt was not a risk factor for hearing loss, but CDDP was.

Considering the impact of hearing loss, even if subclinical, on the linguistic, pedagogical, and cognitive development of children treated for cancer in childhood,^32^ and considering also that studies demonstrating a significant otoprotective effect have not yet been published,^33^ the most important tool in the follow-up of these patients is certainly monitoring.^34^

## Conclusion

The hearing loss identified in cancer patients, examined years after the completion of treatment was sensorineural, bilateral, and symmetrical, and predominantly affected the frequencies of 4, 6, and 8 kHz.

Chemotherapy with CDDP was demonstrated to be a risk factor for acquisition of hearing loss, while head and neck radiation therapy was not decisive.

## Conflicts of interest

The authors declare no conflicts of interest.
